# Supplementary material for: Staging dementia based on caregiver reported patient symptoms: Implications from a latent class analysis
Source: PLoS One. 2020 Jan 15;15(1):e0227857. doi: 10.1371/journal.pone.0227857 (PMC6961931; doi:10.1371/journal.pone.0227857)
Supplement: Attachment — Submitted filename: Response to Reviewers.docx [file pone.0227857.s001.docx]

6^th^ Dec 2019

MS ID: PONE-D-19-30761
MS title: Staging Dementia based on Caregiver Reported Patient Symptoms: Implications from a Latent Class Analysis

Dear reviewers,

Thank you very much for the review and also for the valuable comments and suggestions. Our point-by-point responses are provided below and in bold for easy reference, and highlighted with track changes in the revised manuscript.

We hope that these changes are satisfactory. Thank you very much again in advance!

Sincerely,

Corresponding author

Journal Requirements:

When submitting your revision, we need you to address these additional requirements.

1. Please ensure that your manuscript meets PLOS ONE's style requirements, including those for file naming. The PLOS ONE style templates can be found at <https://imsva91-ctp.trendmicro.com:443/wis/clicktime/v1/query?url=http%3a%2f%2fwww.journals.plos.org%2fplosone%2fs%2ffile%3fid%3dwjVg%2fPLOSOne%5fformatting%5fsample%5fmain%5fbody.pdf&umid=4913B737-987A-2E05-A0D2-C3CB71FD25E2&auth=6e3fe59570831a389716849e93b5d483c90c3fe4-02f1250312cbd8cc3126c5bcb57b48446f45e8ae> and <https://imsva91-ctp.trendmicro.com:443/wis/clicktime/v1/query?url=http%3a%2f%2fwww.journals.plos.org%2fplosone%2fs%2ffile%3fid%3dba62%2fPLOSOne%5fformatting%5fsample%5ftitle%5fauthors%5faffiliations.pdf&umid=4913B737-987A-2E05-A0D2-C3CB71FD25E2&auth=6e3fe59570831a389716849e93b5d483c90c3fe4-cf0dcc3759a9e88581514f7555151ea3ba18bb04>

**RESPONSE: The manuscript is formatted following PLOS ONE style template.**

2. Please refer to any sample size calculations performed prior to participant recruitment. If these were not performed please justify the reasons. Please refer to our statistical reporting guidelines for assistance (<https://imsva91-ctp.trendmicro.com:443/wis/clicktime/v1/query?url=https%3a%2f%2fjournals.plos.org%2fplosone%2fs%2fsubmission%2dguidelines.%23loc%2dstatistical%2dreporting&umid=4913B737-987A-2E05-A0D2-C3CB71FD25E2&auth=6e3fe59570831a389716849e93b5d483c90c3fe4-bc31dbd4e458bf3b4e0e8dc40582663843766e1d>).

**RESPONSE: We did calculate the sample size prior to initiating the study. However, since the calculation was based on the main project outcome (i.e. prevalence of depression), the current study is an exploratory analysis based on the sample collected, which is different from the main project outcome. In this case, we thought it would be misleading if we include the sample size calculation (based on main project outcome) in the manuscript.**

3. We note that you have indicated that data from this study are available upon request. PLOS only allows data to be available upon request if there are legal or ethical restrictions on sharing data publicly. For information on unacceptable data access restrictions, please see https://imsva91-ctp.trendmicro.com:443/wis/clicktime/v1/query?url=http%3a%2f%2fjournals.plos.org%2fplosone%2fs%2fdata%2davailability%23loc%2dunacceptable%2ddata%2daccess%2drestrictions&umid=4913B737-987A-2E05-A0D2-C3CB71FD25E2&auth=6e3fe59570831a389716849e93b5d483c90c3fe4-60195c19acf64d4762b069957f2ef692d6956ca7.

In your revised cover letter, please address the following prompts:

a) If there are ethical or legal restrictions on sharing a de-identified data set, please explain them in detail (e.g., data contain potentially identifying or sensitive patient information) and who has imposed them (e.g., an ethics committee). Please also provide contact information for a data access committee, ethics committee, or other institutional body to which data requests may be sent.

b) If there are no restrictions, please upload the minimal anonymized data set necessary to replicate your study findings as either Supporting Information files or to a stable, public repository and provide us with the relevant URLs, DOIs, or accession numbers. Please see https://imsva91-ctp.trendmicro.com:443/wis/clicktime/v1/query?url=http%3a%2f%2fwww.bmj.com%2fcontent%2f340%2fbmj.c181.long&umid=4913B737-987A-2E05-A0D2-C3CB71FD25E2&auth=6e3fe59570831a389716849e93b5d483c90c3fe4-5482821ba37a53710fcbddbac167cae3ed426272 for guidelines on how to de-identify and prepare clinical data for publication. For a list of acceptable repositories, please see https://imsva91-ctp.trendmicro.com:443/wis/clicktime/v1/query?url=http%3a%2f%2fjournals.plos.org%2fplosone%2fs%2fdata%2davailability%23loc%2drecommended%2drepositories&umid=4913B737-987A-2E05-A0D2-C3CB71FD25E2&auth=6e3fe59570831a389716849e93b5d483c90c3fe4-1305d747dc89a01960ad75d3185e772a533fd7de.

We will update your Data Availability statement on your behalf to reflect the information you provide.

**RESPONSE: The authors' funding agency or government law only permits sharing of human participant data with researchers with whom they have a written agreement. The restrictions have been imposed by our Institutional Review Board (IRB) and Institutional Committee (NHG Domain Specific Review Board and IMH Clinical Research Committee). Our IRB guidelines suggest that a Research Collaboration Agreement (RCA) be signed with collaborating parties. However, data sharing with clear research purposes are available upon request to the senior author. The contact details are included in the cover letter to the editor.**

Additional Editor Comments (if provided):


[Note: HTML markup is below. Please do not edit.]

Reviewers' comments:

Reviewer's Responses to Questions

**Comments to the Author**

1. Is the manuscript technically sound, and do the data support the conclusions?

The manuscript must describe a technically sound piece of scientific research with data that supports the conclusions. Experiments must have been conducted rigorously, with appropriate controls, replication, and sample sizes. The conclusions must be drawn appropriately based on the data presented. 

Reviewer #1: Partly

Reviewer #2: Yes

2. Has the statistical analysis been performed appropriately and rigorously? 

Reviewer #1: Yes

Reviewer #2: I Don't Know

3. Have the authors made all data underlying the findings in their manuscript fully available?

The [PLOS Data policy](https://imsva91-ctp.trendmicro.com/wis/clicktime/v1/query?url=http%3a%2f%2fwww.plosone.org%2fstatic%2fpolicies.action%23sharing&umid=4913B737-987A-2E05-A0D2-C3CB71FD25E2&auth=6e3fe59570831a389716849e93b5d483c90c3fe4-890027d83b7c4ec2eb9c9df5957ec3cb2b18a6b5) requires authors to make all data underlying the findings described in their manuscript fully available without restriction, with rare exception (please refer to the Data Availability Statement in the manuscript PDF file). The data should be provided as part of the manuscript or its supporting information, or deposited to a public repository. For example, in addition to summary statistics, the data points behind means, medians and variance measures should be available. If there are restrictions on publicly sharing data—e.g. participant privacy or use of data from a third party—those must be specified.

Reviewer #1: No

Reviewer #2: No

4. Is the manuscript presented in an intelligible fashion and written in standard English?

PLOS ONE does not copyedit accepted manuscripts, so the language in submitted articles must be clear, correct, and unambiguous. Any typographical or grammatical errors should be corrected at revision, so please note any specific errors here.

Reviewer #1: Yes

Reviewer #2: Yes

5. Review Comments to the Author

Please use the space provided to explain your answers to the questions above. You may also include additional comments for the author, including concerns about dual publication, research ethics, or publication ethics. (Please upload your review as an attachment if it exceeds 20,000 characters)

**Reviewer #1:** The manuscript reports results of staging Dementia based on Caregiver Reported Patient Symptoms: implications from a Latent Class Analysis.

Generally, it aims to stage dementia based on caregiver reported symptoms of persons with dementia. Optimal cut-offs for the assessment tools used were also calculated. The paper is an interesting study that evaluates several specific questions. However, in my opinion there are several aspects should be revised to improve the explanatory power of the manuscript as noted below.

**RESPONSE: Thank you very much!**

Important:

*** Do not use more than 5 abbreviations in the manuscript. After reducing the number of abbreviations, create a table that includes the abbreviations used.

**RESPONSE: We would like to thank the reviewer for this comment. According to the 6^th^ APA guideline, it suggested that abbreviations should be used when the term appears at least three times in the manuscript, and there are no hard lines regarding how many abbreviations are too many. We have revised the manuscript following this guideline, keeping those abbreviations that appears at least three times. And as the reviewer suggested, we have created a list of abbreviations in the end of the manuscript. Please refer to the revised manuscript for more details.**

*** Materials and Methods, Results and Discussion: to facilitate reading I recommend to the authors include a paragraph for each objective.

**RESPONSE: We are sorry that we are not quite sure about what exactly the reviewer is suggesting. In fact, the current manuscript is organized the same way as all other published articles in the journal. May we request that this point be further clarified with an example from any other articles?**

Abstract
Results: Include the main results and p-values if there was significance.

**RESPONSE: For the latent class analysis and the accuracy statistics in the results section of the abstract, both didn’t involve any statistical comparisons, thus there were no relevant p-values. We had made some necessary changes to the results section in the revisions to make it clearer.**

Conclusion: Delete last sentence and include it in the last part of Discusion.

**RESPONSE: The manuscript has been revised accordingly.**

Materials and Methods
a) Participants and Procedures. To create a paragraph entitled: Ethics approval and consent to participate (or similar). I recommend this text:
The protocol was approved by xxxxxx (city, country; with reference number xxxxxxxxxx). The study was developed in accordance with the precepts of the Declaration of Helsinki. All patients signed the corresponding written informed consent prior to their enrolment and appropriate measures were taken to ensure data privacy.

**RESPONSE: In fact, we did have a separate paragraph on the study ethics (i.e. the last paragraph of the ‘Participants and Procedures’ section. The ethics statement has been revised accordingly as suggested by the reviewer.**

b) Measurements
1. Lines 120-122: It is very important to justify why were used 2 scales from 1963 and 1969 respectively to know functional status, memory and behavior problems. Please, explain the reasons why more recent scales were not used.

**RESPONSE: We would like to apologize for not clarifying this in the original manuscript. There are several reasons: first of all, these two scales are both very classical, and there are plenty of articles demonstrating their reliability. Secondly, it has been validated in Singapore (ADL [1, 2] and IADL [3]), and also been used among caregivers in Singapore before, showed good internal reliability [4]. Lastly, it is short and easily administered among dementia caregivers who usually have an average age of 50 years and more.**

2. Line 132: To include reference for the checklist RMBPC.

**RESPONSE: In fact, the reference of RMBPC was included in the manuscript – in the next sentence while describing the checklist. To avoid confusion, we have added in the citation in line 132 (now line 135) as suggested by the reviewer.**

3. Lines 139-140: To explain the reason to use the 20-item Centre for Epidemiological Study Scale from 1977. Please, explain the reason why more recent scale was not used.

**RESPONSE: There are a few reasons: 1) according the American Psychological Association [5], the CES-D provides cut-off scores that aid to identify individuals at risk for clinical depression, with good sensitivity and specificity and high internal consistency [6], and it is sensitive to difference between caregivers and non-caregivers [7], and it can be used across wide age ranges [6]; 2) it has been validated in Singapore before [8]; 3) a previous Singapore study suggested that it has good validity and utility in detecting depression among family caregivers of PWD [9].**

4. Are the used scales validated in Singapur?

**RESPONSE: Please refer to our responses to comments (answers to b) measurement – point 1 and point 3)**

c) Data analysis
Modify this section: One paragraph for every step performed.

**RESPOSE: To make it clearer to the audience, we use numbered list to present the four steps of the analyses. Please refer to the revised manuscript for the details.**

Results
Table 1:
a) No simple size calculations are referred to in this manuscript. Besides, the skewed nature of the simple populaton is not throughly discussed.

b) To diference between 1) mean and frequency 2) SD and percentage.

c) Include a column with p-values.
d) At the table botton include the meaning of SGD and PWD.
e) Tables 2, 3 and 5 include: a) p-values and 2) the meaning of the abreviations.

**RESPONSE: a) We did calculate the sample size prior to initiating the study. However, since the calculation was based on the main project outcome (i.e. prevalence of depression), the current study is an exploratory analysis based on the sample collected, which is different from the main project outcome. In this case, we thought it would be misleading if we include the sample size calculation (based on main project outcome) in the manuscript.**

**Our study sample is informal dementia caregivers, which in nature would differ from the general population. However, this doesn’t necessarily suggest that our sample is skewed. In fact, we compared our sample characteristics with the sample characteristics of dementia caregivers from a previous Singapore Dementia Caregiver Profile study (SDCP) [10], and we found very similar statistics. For example, average age: current study vs. SDCP – 55.7 (11.8) vs. 52.6 (11.1); female proportion: current study vs. SDCP – 75.2% vs. 73.6; relationship with PWD: current study vs. SDCP – 15.3% vs. 18% (spouse), 72.3% vs. 69.2% (children), and 12.4% vs. 12.8% (others); Living with PWD: current study vs. SDCP – 70.2% vs. 79.2%; and having a domestic maid: current study vs. SDCP – 57.1% vs. 52.8%. But we also understand the self-selection bias might affect the generalizability of the study findings, thus this limitation was added.**

**b) Table 1 was re-formatted accordingly.**

**c) Table 1 includes only the descriptive statistics, p-value is not applicable here.**

**d) The meaning of SGD and PWD were added as suggested.**

**e) Table 2 is the latent class analysis results, table 3 and table 5 both included descriptive statistics, all these three tables don’t involve p-values.**

**As suggested by the reviewer, we have added in the meaning of all abbreviations as the notes of the tables.**

Discussion
a) First paragraph: clearly include which are the main findings.
b) I recommend a paragraph for every objective and compare the main results with previous studies.
c) Include a paragraph that includes the main strengths and weaknesses of the study.
**RESPONSE: a) Our main finding is that staging dementia based on caregivers reported is a viable approach which had been included in the first paragraph of the discussion section. However, we do feel the original first paragraph included too many details; thus, we have cut it into two paragraphs so that the new first paragraph is focused on the main study findings, and the following paragraph is focused on the details.**

**b) After the above revisions, the structure of the discussion section follows exactly what the reviewer suggested. In fact, this is the first study which uses such analytical strategies among informal dementia caregivers. There was one study which used artificial intelligence to stage dementia based on caregiver reported patient symptoms, although we had included it in the original manuscript, since the two use quite different methodologies, we felt it was not appropriate for us to directly compare them. Other than that, we cannot identify any other similar previous studies for us to compare.**

**c) We are sorry for the confusion. In fact, the last two paragraphs of the discussion section were the study strengths and limitations. In the revision, we have added a section named ‘Strengths and Limitations’ to avoid confusion.**

**Reviewer #2:** Congratulations on the excellent work. Visiting the important role of informal caregivers is a challenge. I think your study helps to empower the figure of the caregiver of people with dementia.
However, some aspects can be improved when reporting your work in the manuscript.

**RESPONSE: Thanks!**

* Abstract:
I recommend deleting the following text from the "objective" section: "Optimal cut-offs for the assessment tools used were also calculated. “The writing of a research objective should not include a sentence about a result or method that has been performed, but what is intended to be discovered with the study.

**RESPONSE: We would like to thank the reviewer for this comment, the abstract was revised accordingly.**

* The introduction section is well structured, with a good justification for the study and an adequate background.

**RESPONSE: Thanks!**

* I understand that no probabilistic sampling has been performed and this should be explained both in the method section and in the limitations of the study in the discussion section.

**RESPONSE: We are sorry for the negligence. We have added in some descriptions on the non-probabilistic sampling in the methods and study limitations sections, please refer to the revised manuscript for more details.**

* More specific information about the ethical issues of the study would be necessary. The ethical aspects are not adequately explained and are summarized in that it was approved by a committee.

**RESPONSE: We appreciate the reviewer for this comment. More details regarding the ethics were added as suggested.**

* The results are adequately discussed in the manuscript. The limitations are exposed and that is a strength in scientific research. Congratulations

**RESPONSE: Thanks!**

* The possible future lines of research have already been set out in the discussion section. It is not necessary to repeat them in the conclusion section, so you would delete that text from one section or the other.

**RESPONSE: As suggested by the reviewer, the future lines of research in the ‘Conclusion’ section was removed to avoid duplicity.**

6. PLOS authors have the option to publish the peer review history of their article ([what does this mean?](https://imsva91-ctp.trendmicro.com/wis/clicktime/v1/query?url=https%3a%2f%2fjournals.plos.org%2fplosone%2fs%2feditorial%2dand%2dpeer%2dreview%2dprocess%23loc%2dpeer%2dreview%2dhistory&umid=4913B737-987A-2E05-A0D2-C3CB71FD25E2&auth=6e3fe59570831a389716849e93b5d483c90c3fe4-d8012d56528fb7ed61acdf62636337baa20f3ed0)). If published, this will include your full peer review and any attached files.


If you choose “no”, your identity will remain anonymous but your review may still be made public.


**Do you want your identity to be public for this peer review?** For information about this choice, including consent withdrawal, please see our [Privacy Policy](https://imsva91-ctp.trendmicro.com/wis/clicktime/v1/query?url=https%3a%2f%2fwww.plos.org%2fprivacy%2dpolicy&umid=4913B737-987A-2E05-A0D2-C3CB71FD25E2&auth=6e3fe59570831a389716849e93b5d483c90c3fe4-82896d265caab0029f0683c96bc5b010671c9e4c).

Reviewer #1: No

Reviewer #2: No


[NOTE: If reviewer comments were submitted as an attachment file, they will be attached to this email and accessible via the submission site. Please log into your account, locate the manuscript record, and check for the action link "View Attachments". If this link does not appear, there are no attachment files to be viewed.]


While revising your submission, please upload your figure files to the Preflight Analysis and Conversion Engine (PACE) digital diagnostic tool, [https://pacev2.apexcovantage.com/](https://imsva91-ctp.trendmicro.com/wis/clicktime/v1/query?url=https%3a%2f%2fpacev2.apexcovantage.com&umid=4913B737-987A-2E05-A0D2-C3CB71FD25E2&auth=6e3fe59570831a389716849e93b5d483c90c3fe4-aeb3aa5c0ed7b333bbee4e8540f38ff31b57da61). PACE helps ensure that figures meet PLOS requirements. To use PACE, you must first register as a user. Registration is free. Then, login and navigate to the UPLOAD tab, where you will find detailed instructions on how to use the tool. If you encounter any issues or have any questions when using PACE, please email us at [figures@plos.org](mailto:figures@plos.org). Please note that Supporting Information files do not need this step.

**References**

1. Sien NY, Jung HJTSFP. Assessment of the six activities of daily living in adults. 2014;40(4):26-36.

2. Xie F, Li S-C, Roos EM, Fong K-Y, Lo N-N, Yeo S-J, et al. Cross-cultural adaptation and validation of Singapore English and Chinese versions of the Knee injury and Osteoarthritis Outcome Score (KOOS) in Asians with knee osteoarthritis in Singapore. 2006;14(11):1098-103.

3. Ng T-P, Niti M, Chiam P-C, Kua E-HJTJoGSABS, Sciences M. Physical and cognitive domains of the instrumental activities of daily living: validation in a multiethnic population of Asian older adults. 2006;61(7):726-35.

4. Østbye T, Malhotra R, Malhotra C, Arambepola C, Chan AJJoGSBPS, Sciences S. Does support from foreign domestic workers decrease the negative impact of informal caregiving? Results from Singapore survey on informal caregiving. 2013;68(4):609-21.

5. The American Psycholiglcal Association. Center for Epidemiological Studies-Depression [cited 2019 2 Dec]. Available from: <https://www.apa.org/pi/about/publications/caregivers/practice-settings/assessment/tools/depression-scale>.

6. Lewinsohn PM, Seeley JR, Roberts RE, Allen NBJP, aging. Center for Epidemiologic Studies Depression Scale (CES-D) as a screening instrument for depression among community-residing older adults. 1997;12(2):277.

7. Pinquart M, Sörensen SJP, aging. Differences between caregivers and noncaregivers in psychological health and physical health: a meta-analysis. 2003;18(2):250.

8. Stahl D, Sum CF, Lum SS, Liow PH, Chan YH, Verma S, et al. Screening for depressive symptoms: validation of the center for epidemiologic studies depression scale (CES-D) in a multiethnic group of patients with diabetes in Singapore. 2008;31(6):1118-9.

9. Ying J, Yap P, Gandhi M, Liew TMJD, disorders gc. Validity and utility of the center for epidemiological studies depression scale for detecting depression in family caregivers of persons with dementia. 2019;47(4-6):323-34.

10. Tew CW, Tan LF, Luo N, Ng WY, Yap PJD, Disorders GC. Why family caregivers choose to institutionalize a loved one with dementia: a Singapore perspective. 2010;30(6):509-16.
