# Supplementary material for: Staging dementia based on caregiver reported patient symptoms: Implications from a latent class analysis
Source: PLoS One. 2020 Jan 15;15(1):e0227857. doi: 10.1371/journal.pone.0227857 (PMC6961931; doi:10.1371/journal.pone.0227857)
Supplement: Attachment — Submitted filename: Response to Reviewers.docx [file pone.0227857.s002.docx]

27^th^ Dec 2019

MS ID: PONE-D-19-30761R1
MS title: Staging Dementia based on Caregiver Reported Patient Symptoms: Implications from a Latent Class Analysis

Dear reviewers,

Thank you very much for the review and also for the valuable comments and suggestions. Our point-by-point responses are provided below and in bold for easy reference, and highlighted with track changes in the revised manuscript.

We hope that these changes are satisfactory. Thank you very much again in advance!

Sincerely,

Corresponding author

**Comments to the Author**

1. If the authors have adequately addressed your comments raised in a previous round of review and you feel that this manuscript is now acceptable for publication, you may indicate that here to bypass the “Comments to the Author” section, enter your conflict of interest statement in the “Confidential to Editor” section, and submit your "Accept" recommendation.

Reviewer #1: All comments have been addressed

Reviewer #2: (No Response)

**RESPONSE: Thanks!**

2. Is the manuscript technically sound, and do the data support the conclusions?

The manuscript must describe a technically sound piece of scientific research with data that supports the conclusions. Experiments must have been conducted rigorously, with appropriate controls, replication, and sample sizes. The conclusions must be drawn appropriately based on the data presented. 

Reviewer #1: Yes

Reviewer #2: (No Response)

3. Has the statistical analysis been performed appropriately and rigorously? 

Reviewer #1: Yes

Reviewer #2: (No Response)

4. Have the authors made all data underlying the findings in their manuscript fully available?

The [PLOS Data policy](https://imsva91-ctp.trendmicro.com/wis/clicktime/v1/query?url=http%3a%2f%2fwww.plosone.org%2fstatic%2fpolicies.action%23sharing&umid=5B2E0BE8-9A6F-FA05-8C12-4E2DD983887B&auth=6e3fe59570831a389716849e93b5d483c90c3fe4-e19dfa981a5e5fdd5701a4b3dbd05010283da65f) requires authors to make all data underlying the findings described in their manuscript fully available without restriction, with rare exception (please refer to the Data Availability Statement in the manuscript PDF file). The data should be provided as part of the manuscript or its supporting information, or deposited to a public repository. For example, in addition to summary statistics, the data points behind means, medians and variance measures should be available. If there are restrictions on publicly sharing data—e.g. participant privacy or use of data from a third party—those must be specified.

Reviewer #1: Yes

Reviewer #2: (No Response)

5. Is the manuscript presented in an intelligible fashion and written in standard English?

PLOS ONE does not copyedit accepted manuscripts, so the language in submitted articles must be clear, correct, and unambiguous. Any typographical or grammatical errors should be corrected at revision, so please note any specific errors here.

Reviewer #1: Yes

Reviewer #2: (No Response)

6. Review Comments to the Author

Please use the space provided to explain your answers to the questions above. You may also include additional comments for the author, including concerns about dual publication, research ethics, or publication ethics. (Please upload your review as an attachment if it exceeds 20,000 characters)

Reviewer #1: First of all I would like to thank the authors of this manuscript for having addressed the issues raised previously (I think the article has been greatly improved, in my opinion).

**RESPOSE: Thank you very much!**

I just have to make two points:

1) Table 1: in order that the results can be compared to other studies I recommend converting the currency "Singapore dollars" to "Euros or American dollars".

**RESPONSE: We would like to thank the reviewer for this comment. In the revision, we have added in the income level as converted into US dollars to enable easier comparisons with international studies.**

2) Strengths and limitations: delete paragraph 1 and put it at the end of the Discussion.

**RESPONSE: We would like to clarify – in fact that section is about strengths and limitations. The first paragraph is on strengths and the second paragraph is on limitations. In this case, it makes more sense for us to keep the current structure.**

Thank you,

Reviewer #2: (No Response)

7. PLOS authors have the option to publish the peer review history of their article ([what does this mean?](https://imsva91-ctp.trendmicro.com/wis/clicktime/v1/query?url=https%3a%2f%2fjournals.plos.org%2fplosone%2fs%2feditorial%2dand%2dpeer%2dreview%2dprocess%23loc%2dpeer%2dreview%2dhistory&umid=5B2E0BE8-9A6F-FA05-8C12-4E2DD983887B&auth=6e3fe59570831a389716849e93b5d483c90c3fe4-411a624780065cc07aa7edb421091b72b6eb3d14)). If published, this will include your full peer review and any attached files.


If you choose “no”, your identity will remain anonymous but your review may still be made public.


**Do you want your identity to be public for this peer review?** For information about this choice, including consent withdrawal, please see our [Privacy Policy](https://imsva91-ctp.trendmicro.com/wis/clicktime/v1/query?url=https%3a%2f%2fwww.plos.org%2fprivacy%2dpolicy&umid=5B2E0BE8-9A6F-FA05-8C12-4E2DD983887B&auth=6e3fe59570831a389716849e93b5d483c90c3fe4-ba5bd1878bf5c11925b8bc7db36ab03ba6638625).

Reviewer #1: No

Reviewer #2: No


[NOTE: If reviewer comments were submitted as an attachment file, they will be attached to this email and accessible via the submission site. Please log into your account, locate the manuscript record, and check for the action link "View Attachments". If this link does not appear, there are no attachment files to be viewed.]


While revising your submission, please upload your figure files to the Preflight Analysis and Conversion Engine (PACE) digital diagnostic tool, [https://pacev2.apexcovantage.com/](https://imsva91-ctp.trendmicro.com/wis/clicktime/v1/query?url=https%3a%2f%2fpacev2.apexcovantage.com&umid=5B2E0BE8-9A6F-FA05-8C12-4E2DD983887B&auth=6e3fe59570831a389716849e93b5d483c90c3fe4-9d50f47631ec1eec6f55c39214daf2416edc9579). PACE helps ensure that figures meet PLOS requirements. To use PACE, you must first register as a user. Registration is free. Then, login and navigate to the UPLOAD tab, where you will find detailed instructions on how to use the tool. If you encounter any issues or have any questions when using PACE, please email us at [figures@plos.org](mailto:figures@plos.org). Please note that Supporting Information files do not need this step.
